# Trop-2-Expression in soliden Tumoren: Erweiterte Proteindiagnostik im Rahmen des Molekularen Tumorboards

**DOI:** 10.1007/s00292-025-01441-x

**Published:** 2025-06-27

**Authors:** Elaine-Pashupati Dopfer, Matthias Fahrner, Johanna Thiery, Stepan Riemer Cysar, Jannis Heyer, Peter Bronsert, Heiko Becker, Julia C. Kuehn, Theresa Lowinus, Linda Gräßel, Justyna Rawluk, Melanie Boerries, Maryam Barsch, Michael Quante, Christoph Peters, Justus Duyster, Anne Schultheis, Martin Werner, Christoph Cornelius Miething, Oliver Schilling, Sylvia Timme-Bronsert

**Affiliations:** 1 Institut für Klinische Pathologie, Universitätsklinikum Freiburg, Breisacher Str. 115a, 79106 Freiburg, Deutschland; 2 Comprehensive Cancer Center Freiburg (CCCF), Medizinische Fakultät, Universitätsklinikum Freiburg, Freiburg, Deutschland; 3 Konsortium für Translationale Krebsforschung (DKTK) und Deutsches Krebsforschungszentrum (DKFZ), Partnerstandort Freiburg, Freiburg, Deutschland; 4 Core Facility für Histopathologie und Digitale Pathologie, Universitätsklinikum Freiburg, Freiburg, Deutschland; 5 Klinik für Innere Medizin I, Hämatologie, Onkologie und Stammzelltransplantation, Universitätsklinikum Freiburg, Freiburg, Deutschland; 6 Institut für Medizinische Bioinformatik und Systemmedizin, Universitätsklinikum Freiburg, Freiburg, Deutschland; 7 Klinik für Innere Medizin II, Gastroenterologie, Hepatologie, Endokrinologie und Infektiologie, Universitätsklinikum Freiburg, Freiburg, Deutschland

**Keywords:** Biomarker, Immunkonjugate, Off-Label-Use, Präzisionsmedizin, Proteomics, Biomarkers, Immunoconjugates, Off-label use, Precision medicine, Proteomics

## Abstract

**Hintergrund:**

„Trophoblast surface antigen 2“ (Trop-2) ist ein Oberflächenantigen, das als Zielstruktur neuer Antikörper-Wirkstoff-Konjugate (ADC) dient. Wir berichten über die Evaluation der Trop-2-Expression und die Bestimmung des Biomarkers in soliden Tumoren im Rahmen einer erweiterten Proteindiagnostik im Molekularen Tumorboard Freiburg (MTB).

**Methodik:**

Die entitätenübergreifende Kohorte umfasst 50 Fälle des Comprehensive Cancer Center Freiburg (CCCF), die im interdisziplinären MTB vorgestellt wurden und für die eine Trop-2-Immunhistochemie (IHC) durchgeführt wurde. Die Trop-2-Expression wurde mittels IHC an formalinfixierten, in Paraffin eingebetteten Gewebeproben bestimmt. Unter Verwendung des H‑Scores wurden die Proben in die Kategorien „negativ“, „niedrige“, „mittlere“ und „hohe“ Expression eingeteilt. Bei 22 Patienten erfolgte zusätzlich eine massenspektrometrische (MS) Trop-2-Analyse sowie eine Korrelationsanalyse der Trop-2-Intensitäten.

**Ergebnisse:**

Die Trop-2-Expression wurde in 16 % der Fälle als negativ, in 20 % der Fälle niedrig, in 18 % der Fälle mäßig und in 46 % der Fälle als hoch bewertet. Die Trop-2-IHC-Scores korrelieren positiv mit den MS-basierten Proteinintensitäten. Behandlungsempfehlungen für ein Trop-2-ADC wurden in 61 % aller Fälle gegeben und Trop-2-ADC wurden in 20 % der Fälle als therapeutischer Ansatz mit der höchsten Priorität bewertet.

**Diskussion:**

Die Trop-2-Bestimmung bietet eine Grundlage für personalisierte Behandlungsempfehlungen, die Priorisierung von Therapieoptionen und den Off-Label-Use im MTB. Die positive Korrelation zwischen den Trop-2-ICH-Werten und MS-basierten Intensitäten validiert die IHC und weist auf ein zukünftiges Potenzial einer Multi-Omics-Bewertung therapeutischer Biomarker hin.

**Graphic abstract:**

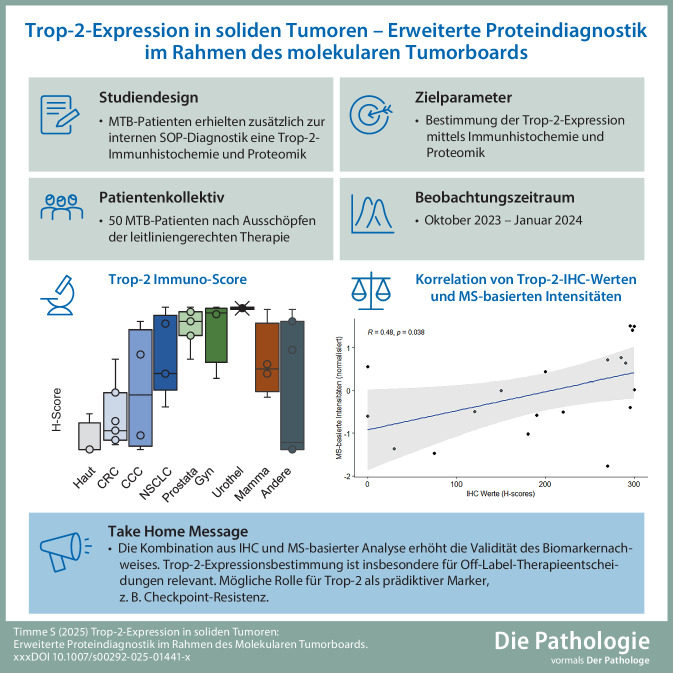

## Abstract (en)

**Background:**

Trophoblast surface antigen 2 (Trop-2) is a surface antigen that can be targeted by novel antibody-drug conjugates (ADCs). Here we report on the systematic evaluation of Trop‑2 expression and the determination of the biomarker in solid tumors as part of expanded protein diagnostics within the molecular tumor board (MTB) in Freiburg.

**Methods:**

The pan-cancer cohort examined comprises 50 patients from the Comprehensive Cancer Center Freiburg who were enrolled in the MTB and additionally received a Trop‑2 IHC staining that was performed using formalin-fixed and paraffin-embedded tissue samples. Utilizing the H‑score, the samples were categorized into negative-, low-, moderate-, or high-expressing tumors. Additionally, mass spectrometry (MS)-based proteomics was performed on 22 tissue samples and used for correlation analysis with IHC expression levels.

**Results:**

Trop‑2 expression was negative in 16%, low in 20%, moderate in 18%, and high in 46% of the assessed cases. The Trop‑2 IHC scores showed a positive correlation with MS-based protein intensities (Pearson correlation coefficient 0.48). Treatment recommendation for Trop‑2 ADC was given in 61% and Trop‑2 ADC was considered as the best therapeutic option in 20%.

**Discussion:**

The evaluation of Trop‑2 expression might be helpful for personalized treatment recommendations in the off-label setting as well as ranking strategies for personalized therapeutic options within the MTB. The positive correlation between Trop‑2 IHC scores and MS-based protein intensities validates the IHC and demonstrates the promise of multi-omics assessment of therapeutic biomarkers.

Die Entscheidungsfindung für individualisierte Therapien in der palliativen Situation wird durch den Mangel an prädiktiven Biomarkern erschwert. Das Oberflächenantigen Trop‑2 ist eine vielversprechende Zielstruktur innovativer Antikörper-Wirkstoff-Konjugate (ADC). Für Trop‑2 fehlt aktuell eine unabhängige Validierung als entitätenübergreifender Biomarker und Entscheidungsparameter für In- und Off-Label-Therapien. Diese Studie untersucht die Trop‑2 Expression mit 2 unabhängigen Verfahren (Immunhistochemie und Massenspektrometrie) und diskutiert den Nutzen der Biomarkerbestimmung in soliden Tumoren im Rahmen einer erweiterten Proteindiagnostik innerhalb des Molekularen Tumorboard Freiburg (MTB Freiburg).

## Hintergrund und Fragestellung

Trop‑2 ist ein Transmembranprotein mit einer essenziellen Rolle in der Embryogenese und wird im Kontext der Dedifferenzierung bei Karzinomen überexprimiert. Eine Überexpression von Trop‑2 wurde beispielsweise bei triple-negativem Brustkrebs (TNBC), Urothelkarzinomen, nichtkleinzelligem Lungenkrebs (NSCLC) und Plattenepithelkarzinomen (z. B. primären oralen, vulvären und penilen Karzinomen) beschrieben [7, 13, 14, 23]. Die membranäre Trop-2-Expression korreliert mit den gemessenen mRNA-Leveln [9].

Wesentliche intrazelluläre Trop-2-abhängige Signalkaskaden sind Ca^2+^-vermittelt und umfassen u. a. PI3K/AKT, JAK/STAT, MAPK [16] und die β‑Catenin-Signalkaskaden. Diese haben einen zentralen Stellenwert in onkogenen Prozessen wie Proliferation, Zellmigration [10] sowie bei der Resistenzbildung [15].

Die therapeutische Wirkungsweise von Trop-2-gerichteten ADCs basiert auf rezeptorabhängiger Internalisierung des Antikörper-Wirkstoff-Konjugats und ist weitgehend unabhängig von Trop‑2 vermittelten intrazellulären Signalkaskaden.

**Abb. 1:**
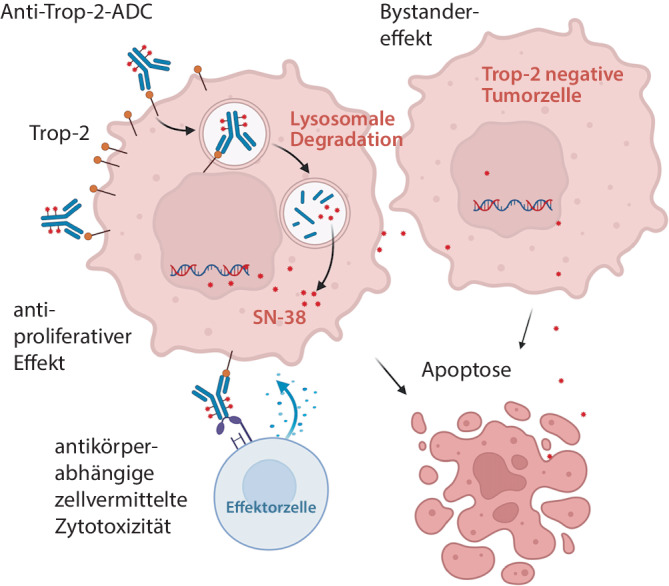
Wirkungsweise von Trop-2-Antikörper-Wirkstoff-Konjugaten. Die spezifische Bindung von Antikörper-Wirkstoff-Konjugaten (*ADC*) an Trop-2-positive Tumorzellen führt zur Endozytose. Nach Abbau und Freisetzung aus den Lysosomen führt das Chemotherapeutikum zu DNA-Schäden, die zur Apoptose der Zielzelle führen. Über einen Bystandereffekt können möglicherweise auch Trop-2-negative Tumorzellen eliminiert werden. MTB SOP (Erstellt in BioRender, Schilling, O. (2025) https://BioRender.com/g47p805)

Neuartige Trop-2-gerichtete ADCs wie Sacituzumab-Govitecan oder Datopotamab-Deruxtecan (DS-1062) sind mit Topoisomerase-I-Hemmern gekoppelt. Diese schädigen nach der spezifischen Aufnahme durch Trop-2-exprimierende Tumorzellen die DNA der Zielzelle. Zudem wurde bei einigen ADCs ein Bystandereffekt beschrieben, sodass auch Biomarker-negative Tumorzellen eliminiert werden könnten (Abb. 1). Ob ein direkter antiproliferativer Effekt über das Trop-2-gerichtete ADC oder ein antikörperabhängiger zytotoxischer Effekt (ADCC) ausgelöst wird, ist Gegenstand aktueller Forschung [15].

Das ADC Sacituzumab-Govitecan ist in Europa bei nichtresezierbarem TNBC [2, 3] und HER2-negativem metastasierten Mammakarzinom (HR+/HER2− mBC) in späteren Therapielinien ohne verpflichtenden Nachweis einer Trop-2-Expression mittels Companion Diagnostic zugelassen.

## Studiendesign und Untersuchungsmethoden

### Probenkollektiv

Die untersuchte Kohorte umfasst die ersten 50 Patienten (*n* = 50), die im Zeitraum von Oktober 2023 bis Januar 2024 im MTB Freiburg des CCCF eine Trop-2-Immunhistochemie erhalten haben. Alle Patienten litten an einer progredienten Tumorerkrankung nach Ausschöpfen der leitliniengerechten Therapie oder dies war absehbar (MTB Freiburg Registerstudie; Ethikvotum 369/19). Bei allen Patienten wurde die intern festgelegte leitlinienkonforme histopathologische Diagnostik bestehend aus entitätenspezifischen IHC(Immunhistochemie)-Färbungen und Next-Generation-Sequencing(NGS)-Panel-basierter molekularer Diagnostik (TruSight Oncology 500 [TSO 500], Illumina, San Diego, USA; Whole Exome Sequencing [WES], RNA-Sequenzierung, RNA-Fusionspanels und/oder DKTK MASTER, NCT Heidelberg, Heidelberg, Deutschland) durchgeführt.

### Trop-2-Immunhistochemie

Die Trop-2-IHC-Färbung wurde an formalinfixierten und in Paraffin eingebetteten (FFPE) Gewebeproben mit dem Trop-2-Antikörperklon EPR20043 (Abcam, Cambridge, UK) und der Verwendung von externen Positivkontrollen mittels DAKO Autostainer Link 48 (Agilent Technologies, Santa Clara, CA, USA) etabliert (Verdünnung 1:2000, Inkubationszeit 20 min bei Raumtemperatur; Antigen Retrieval: 15 min bei 95 °C). H‑Scores wurden mit der folgenden Formel berechnet: H‑Score = (3 × % Zellen mit starker Färbung) + (2 × % Zellen mit mäßiger Färbung) + (1 × % Zellen mit schwacher Färbung). Die Proben wurden als negativ (H-Score 0), niedrige Expression (H-Score 1–99), moderate Expression (H-Score 100–199) oder starke Expression (H-Score 200–300) klassifiziert [5].

### Massenspektrometriebasierte Proteomik

Bei ausreichend vorhandenem Tumorgewebe wurde im Anschluss zur Standard-Operating-Procedures(SOP)-Diagnostik eine massenspektrometriebasierte proteomische Analyse durchgeführt (*n* = 20). Die Proteinextraktion und Solubilisierung erfolgte über 2 Zyklen hitzeinduzierter Antigendemaskierung (HIAR) bei 95 °C für 1 h sowie ultraschallbasierter Gewebehomogenisierung (Bioruptor, Diagenode, Liege, Belgien; 20 Zyklen 30/30 s an/aus). Die Proteinkonzentration wurde mittels Bicinchoninsäure-Assay (Thermo Fisher Scientific, Waltham, MA, USA) bestimmt. Die Probenvorbereitung erfolgte automatisiert via SP3 im 96-Well-Format (Pipettierroboter Bravo, Agilent Technologies, Santa Clara, CA, USA) [19]. Nach Reduktion und Alkylierung wurden die Proteine mit Trypsin (1:25) und LysC (1:100) verdaut und Peptidkonzentrationen bestimmt. Es wurden 300 ng Peptide auf einer 15-cm-Nano-Kapillarsäule (Evosep Biosystems, Odense, Dänemark) getrennt und im Massenspektrometer (timsTOF Flex, Bruker Corporation, Billerica, MA, USA) mittels datenunabhängiger Akquisition (DIA) analysiert. Die Datenauswertung erfolgte mit der EBI-Proteindatenbank (EMBL’s European Bioinformatics Institute [EMBL-EBI], Hinxton, UK; Stand: März 2022) und DIA-NN (v1.8.1) bei einer 1 % False-Discovery-Rate (FDR) auf Protein- und Peptidebene. Zur Proteinidentifikation wurden ausschließlich eindeutig zuweisbare Peptide berücksichtigt. Ergebnisse wurden log2-transformiert, Median-normalisiert und Trop-2-Intensitäten für die Korrelationsanalyse Z‑score-normalisiert.

## Ergebnisse

**Abb. 2:**
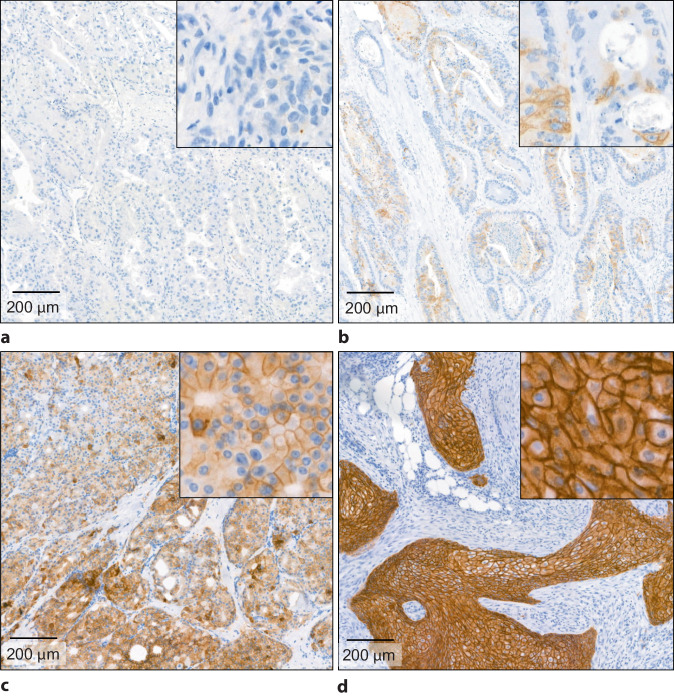
Trop-2-Immunhistochemie. **a** Negativ, Nierenzellkarzinom. **b** Niedrige Expression (H-Score 1–100), Kolonkarzinom. **c** Mäßige Expression (H-Score 100–199), Prostatakarzinom. **d** Hohe Expression (H-Score 200–300), Zervixkarzinom

Die Trop-2-Färbung erfüllte in allen 50 untersuchten FFPE-Gewebeproben die Qualitätskriterien gemäß einer externen Positivkontrolle. Exemplarische Färbungen sind in Abb. 2 dargestellt.

**Abb. 3:**
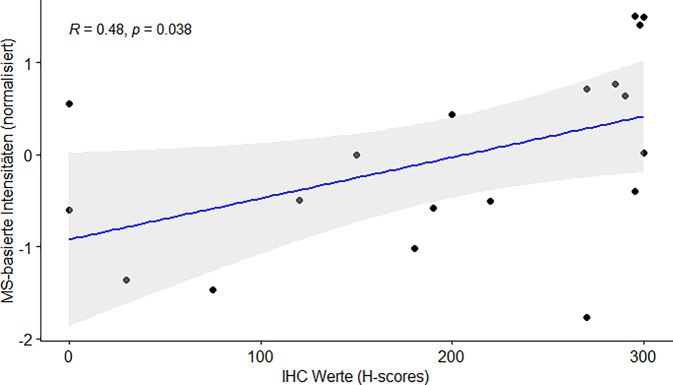
Korrelationsanalyse der Trop-2-H-Scores mit massenspektrometriebasierten Trop-2-Intensitäten. *MS* Massenspektrometrie, *IHC* Immunhistochemie

In 22 Fällen wurde parallel eine MS-basierte proteomische Analyse durchgeführt, wobei in 19 Fällen Trop‑2 identifiziert und quantifiziert wurde. Es zeigte sich eine positive Korrelation der erhobenen IHC-Werte für Trop‑2 mit den Intensitäten in der MS-basierten Analyse (Pearson-Korrelationskoeffizient von 0,48; Abb. 3).

**Abb. 4:**
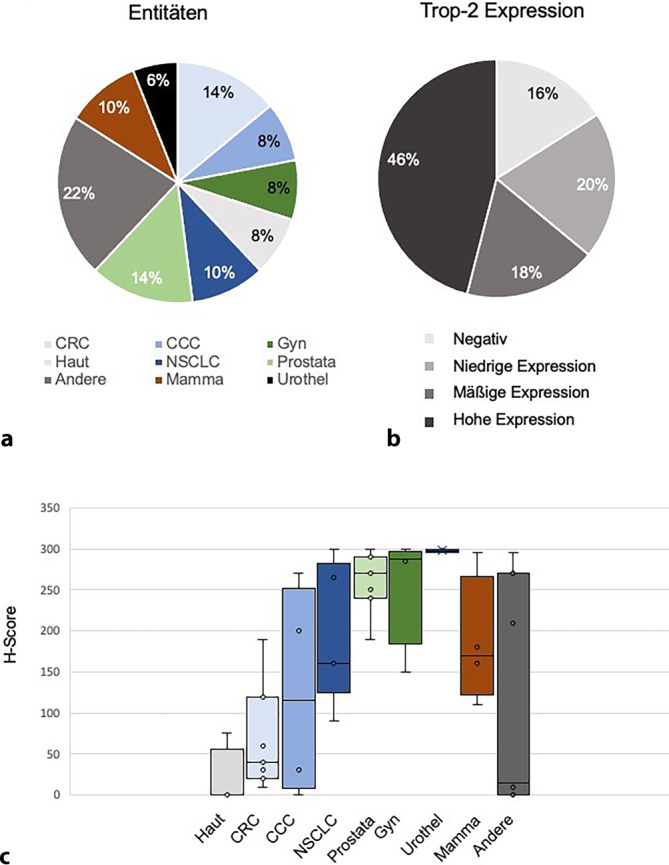
**a** Zusammensetzung der Kohorte gruppiert nach Entitäten. **b** Trop-2-Immunoscore über alle Entitäten. Negativ = H‑Score 0, niedrige Expression = H‑Score 1–99, moderate Expression = H‑Score 100–199, hohe Expression = H‑Score 200–300 **c** Zusammensetzung der Kohorte nach Entitätengruppen mit kontinuierlichen H‑Scores. *CCC* cholangiozelluläres Karzinom, *CRC* kolorektales Karzinom, *Gyn* gynäkologisches Karzinom, *NSCLC* nichtkleinzelliges Lungenkarzinom

In der IHC wurde die Trop-2-Expression in 16 % (*n* = 8) der Fälle als negativ, in 20 % (*n* = 10) der Fälle als niedrig, in 18 % (*n* = 9) der Fälle als mäßig und in 46 % (*n* = 23) der Fälle als hoch bewertet (Abb. 4). Mammakarzinome und urotheliale Karzinome zeigten durchgängig eine hohe oder mittlere Expression. Auch Adenokarzinome der Prostata sowie gynäkologische Tumoren zeigten häufig eine hohe Expression. Neoplasien der Haut blieben zum Großteil Trop-2-negativ, während kolorektale Karzinome überwiegend eine niedrige Trop-2-Expression aufwiesen. Gastrointestinale Tumoren (z. B. cholangiozelluläre Karzinome) zeigten in der kleinen Stichprobe eine hohe interindividuelle Variabilität in der Trop-2-Expression. Insgesamt korrelierten die Expressionslevel der verschiedenen Entitäten, soweit bekannt, mit den Daten aus der Fachliteratur (Abb. 4).

Von den 50 untersuchten Patienten wurden 46 Patienten (*n* = 46) nach Durchführung einer Trop-2-IHC erneut im Molekularen Tumorboard vorgestellt (die übrigen Patienten wünschten keine weitere Therapie oder waren zwischenzeitlich verstorben).

Neben den Ergebnissen aus der genetischen Charakterisierung der Tumore flossen die IHC-Ergebnisse verschiedener ADC-Zielstrukturen in die Therapieempfehlungen mit ein. Therapieempfehlungen für Trop-2-ADC wurden in 59 % (*n* = 27) der wieder vorgestellten Fälle gegeben. Trop-2-ADC wurde in 20 % (*n* = 9) der Fälle als vorrangige therapeutische Option bewertet (Ranking der Therapieoptionen Platz 1). In 2 Fällen (ein TNBC und ein Lungenadenokarzinom) wurde die Therapieempfehlung für Sacituzumab-Govitecan basierend auf m1A-Evidenzleveln vor Februar 2025 umgesetzt (Tab. 1). Bei den übrigen Patienten wurde Sacituzumab-Govitecan erst in späteren Therapielinien vorgeschlagen (Tab. 1, s. Ranking) oder sie waren zwischenzeitlich verstorben (NA) oder wünschten keine weitere Therapie (NA).

**Tab. 1:** Therapieempfehlungen des Molekularen Tumorboards für Trop-2-Antikörper-Wirkstoff-Konjugate bei unterschiedlichen Entitäten

	Expressionslevel	H‑Score	Empfehlung (*n* = x)	Ranking	Therapie S‑G
**CRC**	Niedrig: 5	40, 10, 30,20,60	1	3.	Nein
(*n* = 7)	Mäßig: 2	120,19	Keine	–	–
**CCC**	Negativ: 1	0	Keine	–	–
(*n* = 4)	Niedrig: 1	30	Keine	–	–
	Hoch: 2	200, 270	2	2., 1.	Nein
**Mamma**	Mäßig: 3	110,160, 180	2 (1NA)	2.,2.	Nein
(*n* = 5)	Hoch: 2	295, *290*	2	1., 2.	Nein/*ja*
**Gyn**	Mäßig: 1	150	1	2.	Nein
(*n* = 4)	Hoch: 3	285, 290, 300	3	2., 2., 2.	Nein
**Haut**	Negativ: 3	0,0,0	Keine	–	–
(*n* = 4)	Niedrig: 1	75	Keine	–	–
**NSCLC**	Niedrig: 1	90	Keine	–	–
(*n* = 5)	Mäßig: 2	160, 160	2	1., 1.	Nein
	Hoch: 2	265, *300*	2	1., 1.	Nein/*ja*
**Prostata**	Mäßig: 1	190	1	3.	Nein
(*n* = 7)	Hoch: 6	240, 250, 270	6	2., 3., 4.	Nein
		270, 290, 300		3.,4.	Nein
**Urothel**	Hoch: 3	295, 2 × 300	3	1.,2.,2.	Nein
(*n* = 3)	–	–	–	–	–
**Andere**	Negativ: 4	0, 0, 0, 0	Keine	–	–
(*n* = 11)	Niedrig: 2	10, 15	Keine	–	–
–	Hoch: 4	210, 3 × 270, 295	3 (1 NA)	–	Nein

*CRC* kolorektale Karzinome, *CCC* cholangiozelluläre Karzinome, *Mamma* Mammakarzinome*, Gyn* Gynäkologische Karzinome, *Haut* Karzinome der Haut*, NSCLC* nichtkleinzelliges Lungenkarzinom, *Urothel* Urothelkarzinom, *Andere: *CUP („cancer of unknown primary“, 2), Nierenkarzinome (2), Nasennebenhöhlenkarzinome (2), Adenokarzinom des gastroösophagealen Übergangs (1), Hodenkarzinom (1), neuroendokrines Karzinom (1), Peniskarzinom (1), anaplastisches Schilddrüsenkarzinom (1); *NA* verstorben oder kein weiterer Therapiewunsch

## Diskussion

Die klinische Entscheidung für eine individualisierte Therapie wird zunehmend komplexer. Häufig werden allein auf Grundlage des mittels Sequenzierungsverfahren bestimmten molekularen Profils eines Tumors keine Zielstrukturen identifiziert. Zudem profitieren Patienten von zusätzlichen therapeutischen Optionen. Angesichts wachsender Verfügbarkeit verschiedener ADCs ist eine umfassende Kenntnis der Expression verschiedener Oberflächenproteine als möglicher Biomarker essenziell, wobei der für die ADC-Therapie verwendete Antikörperklon oft nicht für diagnostische Zwecke verfügbar ist. Auch das immunogene Epitop des Proteins ist bei vielen Antikörpern ein „trade secret“. In der vorliegenden Studie wurde die Trop-2-IHC mit einer antikörperunabhängigen MS-basierten Trop-2-Detektion kombiniert und validiert. Ein Vorteil der Massenspektrometrie besteht darin, dass nicht nur einzelne Epitope, sondern mehrere Peptide des Proteins detektiert werden. Eine umfassende Abdeckung des Trop-2-Proteins kann somit für Epitop-regulierende Prozesse wie etwa „shedding“ oder proteolytische Spaltung [22] kompensieren. Unterstützend konnten wir eine positive Korrelation der IHC-Werte (basierend auf dem Antikörperklon EPR20043) mit den Ergebnissen der MS-basierten Proteomik zeigen, was auf ein stabiles und repräsentatives Antikörperepitop auch in der vorliegenden heterogenen Kohorte hinweist. Aufgrund der limitierten Fallzahl (*n* = 19) weist die lineare Regression jedoch einen erweiterten Konfidenzbereich auf. Ein kombiniertes Nachweisverfahren von Trop‑2 wurde für Speicheldrüsenkarzinome mit IHC und MALDI-MS bereits gezeigt [24]. Zur Beurteilung der tumorspezifischen sowie der räumlich aufgelösten Befundung (z. B. membranären Expression) von Trop‑2 ist die IHC der Proteomik überlegen. Die kräftige Expression des Biomarkers auf benignen Epithelien oder eine zytoplasmatische Lokalisation kann von der Proteomik nicht aufgelöst werden. *Die beiden Methoden ergänzen sich synergistisch und könnten perspektivisch in spezialisierten Zentren für bestimmte Fragestellungen gemeinsam Anwendung finden.*

Das Trop-2-gerichtete ADC Sacituzumab-Govitecan wurde ohne Companion Diagnostic für die Behandlung von nichtresezierbarem TNBC [3] und HER2−-mBC [4] zugelassen. Diese Entitäten weisen durchgängig eine mittlere oder hohe Trop-2-Expression auf. In einer Studie mit 468 Patienten verbesserte Sacituzumab-Govitecan im TNBC signifikant das progressionsfreie Überleben (5,6 vs. 1,7 Monate) und das Gesamtüberleben (12,1 vs. 6,7 Monate) im Vergleich zur Chemotherapie bei einer objektiven Ansprechrate von 35 % versus 5 % [2]. Zudem konnte gezeigt werden, dass die Dauer des Therapieansprechens positiv mit dem Trop-2-Expressionslevel korreliert [5, 12, 17]. Im NSCLC ist die Datenlage zunächst weniger eindeutig [1, 20], aber auch für hier gibt es Hinweise, dass Subgruppen mit einem H‑Score von > 220 verstärkt von einer ADC-Therapie profitieren [20]. Weiterhin zeigen Trop-2-gerichtete ADCs vielversprechende Studienergebnisse in einigen anderen Entitäten, wie z. B. dem fortgeschrittenen Endometriumkarzinom [21], welches in der hier untersuchten Kohorte durchgängig eine mittlere oder hohe Trop-2-Expression aufwies.

Die FDA-Zulassung für Sacituzumab-Govitecan wurde für das metastasierte Urothelkarzinom (mUC) [8] aufgrund von Nebenwirkungen und eingeschränkter Wirksamkeit zwischenzeitlich zurückgezogen.

Ob die Wirksamkeit von Trop-2-gerichteten ADCs direkt mit dem Expressionslevel korreliert und/oder auf bestimmte Subgruppen beschränkt ist, ist noch nicht abschließend geklärt und es bedarf hierzu weiterer stratifizierender Studien. Der Bystandereffekt oder eine erhöhte Trop-2-Internalisierung könnten beispielsweise für eine geringe Expression des Tumorantigens kompensieren. Bei Trop-2-Negativität ist von einer Wirksamkeit des ADCs nicht auszugehen. Für den Off-Label-Use ist es daher entscheidend, Tumore ohne Trop-2-Expression zu identifizieren, da diese Patienten vermutlich nicht von einer Trop-2-zielgerichteten Therapie profitieren. Dies ist insbesondere für Entitäten mit inkonsistenter Expression, wie z. B. dem cholangiozellulären Karzinom, oder bei CUP-Syndromen relevant.

Als prognostischer Biomarker könnte der Trop-2-IHC auch unabhängig von der ADC-Therapie eine Relevanz zugesprochen werden. Studien zeigen, dass eine hohe Trop-2-Expression mit einer schlechteren Prognose bei verschiedenen Tumorentitäten, darunter Brustkrebs, Magenkrebs, NSCLC und Kolonkarzinom assoziiert ist [11, 18, 25]. In anderen Entitäten, wie z. B. dem Plattenepithelkarzinom des Penis oder der Vulva, scheint die Überexpression interessanterweise mit einer günstigeren Prognose einherzugehen [23].

Auch als prädiktiver immuntherapeutischer Marker könnte die Trop-2-IHC von Nutzen sein. So wurde in einer retrospektiven Studie eine hohe Trop-2-Expression mit einer möglichen Resistenz gegen Immuncheckpoint-Therapien bei NSCLC in Verbindung gebracht [6].

Vor dem Hintergrund einer aufkommenden Vielzahl von molekularen Targets ist es von Bedeutung, dass vor oder zumindest am Anfang der klinischen Implementierung die Notwendigkeit einer Biomarkerbestimmung und die Validität der Biomarker in prospektiven und retrospektiven Studien evaluiert wird.

## Limitationen und Ausblick

Um die Aussagekraft und Relevanz der hier gezeigten Ergebnisse zu erhöhen, sind umfangreichere Untersuchungen und aufbauende Studien notwendig. Hierzu zählen unter anderem:

- Vergrößerung der Kohorte,
- zusätzliche Korrelation der Trop‑2 Expression mit:
  - genomischen Phänotypen der Tumoren,
  - dem therapeutischen Ansprechen auf Trop-2-ADC,
  - dem Ansprechen oder der Resistenz auf Immuncheckpoint-Therapie oder andere Therapien,
- publik machen objektiver Ergebnisse durch unabhängige Non-profit-Organisationen.

## Fazit für die Praxis

- Das Trop-2-Antikörper-Wirkstoff-Konjugat (Trop-2-ADC) Sacituzumab-Govitecan ist aktuell ohne Companion Diagnostic bei triple-negativem Brustkrebs (TNBC) und HER2-negativem metastatischem Brustkrebs (HER2^–^-mBC) in späteren Therapielinien zugelassen.
- Die Immunhistochemie(IHC)-Werte mit dem Anti-Trop-2-Antikörperklon EPR20043 zeigen eine positive Korrelation mit massenspektrometriebasierten Trop-2-Intensitäten und validieren die Trop-2-IHC.
- Bei komplexen Tumorerkrankungen (wie z. B. im Kontext des Molekularen Tumorboards) könnte die Trop-2-Expressionsbestimmung bei Off-Label-Therapieentscheidungen und CUP(„cancer of unknowm primary“)-Syndromen relevant werden.
- Die Expressionsbestimmung von Trop-2 könnte zukünftig eine Rolle als prädiktiver Marker in der personalisierten Medizin spielen (z. B. Checkpoint-Resistenz).
- Offen bleibt, inwieweit das Trop-2-Expressionsniveau mit dem Therapieansprechen auf Trop-2-ADCs korreliert und ob dieses von Entität, molekularen Subgruppen, Treibermutationen oder anderen Biomarkern abhängt.

## Data Availability

Die in dieser Studie erhobenen Datensätze können auf begründete Anfrage beim Korrespondenzautor angefordert werden.
